# Timing, not magnitude, of force may explain sex-dependent risk of ACL injury

**DOI:** 10.1007/s00167-018-4859-9

**Published:** 2018-02-10

**Authors:** Haraldur B. Sigurðsson, Þórarinn Sveinsson, Kristín Briem

**Affiliations:** Department of Physical Therapy, Faculty of Health Sciences, University of Iceland, Hringbraut 31, 101 Reykjavík, Iceland

**Keywords:** Knee, ACL, Biomechanics, Injury prevention, Motion analysis

## Abstract

**Purpose:**

The anterior cruciate ligament is loaded through valgus moment, vertical ground reaction force, and internal rotation moment. The aim of this study was to compare the timing of force peaks during early stance between youth girls and boys.

**Methods:**

One-hundred and twenty-nine team sport athletes aged 9–12 completed a total of 2540 cutting maneuvers captured with an 8-camera motion capture system. Timing of early force peaks was analyzed within 100 ms after ground contact.

**Results:**

Genders showed different mean (95% CI) time to peak valgus—(32 ms (30–33 ms) vs 37 ms (36–38 ms), *P* < 0.001) and time to peak internal rotation moments (36 ms (35–37 ms) vs 38 ms (37–39 ms), *P* = 0.029) but not time to peak vertical ground reaction force [38 ms (37–40 ms) vs 37 ms (36–38 ms, n.s.)]. Girls showed a smaller time between vertical ground reaction force and valgus moment peaks (mean (95% CI) of 1 ms (1–2 ms) vs 7 ms (5–9 ms), *P* < 0.001), and valgus- and internal rotation moment peaks (0 ms (− 2 to 1.0 ms) vs − 5 ms (− 6 to − 3 ms), *P* = 0.0003) but not between internal rotation moment and vertical ground reaction force.

**Conclusions:**

Concurrent force peaks are more common for girls compared with boys, leading to more frequent multi-planar loading of the knee. Timing may explain sex-dependent risk of ACL injuries. Exposure to repeated cutting movements may result in greater ACL injury risk due to timing of knee forces as well as magnitude. Such exposure should be minimized for at-risk athletes.

**Level of evidence:**

III.

## Introduction

One of the more serious knee injuries that team sports athletes suffer are anterior cruciate ligament (ACL) injuries which can result in significant time loss for the athlete [26], decreased sports performance and career longevity for adult athletes [11], high risk of re-rupture [27], and rapid progression of knee osteoarthritis [1]. The result is a high economic price tag on every injury [20] and decreased long-term knee-related quality of life [8]. Young athletes who sustain an ACL injury have been found to return prematurely to sports [3] with functional deficits persisting for years after injury [10]. The incidence of ACL injuries in soccer matches has been reported to be 0.2 for every 1000 h for men, and up to three times higher for females [36]. Females and young athletes have an increased risk of sustaining a contralateral ACL injury following the first injury [33], and recent reports indicate that frequency of ACL injuries in athletes aged 5–14 years is increasing [29].

The injury is most often a non-contact injury during a landing or cutting maneuver [4]. Cadaver studies, simulating relevant knee joint forces, have demonstrated that the ACL is primarily loaded through anterior tibial translation in relation to the femur [17], external knee valgus moment (VM) and external tibial internal (medial) rotation moment (IRM) [18]. Tibiofemoral joint compression [24] results in anterior tibial translation and is thus a secondary ACL loading mechanism. The resulting load on the ACL is additionally dependent on the knee joint position, most notably the flexion angle [17, 19], and anatomic factors such as the tibial slope [23, 37] and ACL structural properties, both of which are sex-dependant [22, 38]. A combination of forces results in greater strain on the ACL than individual forces [2, 12, 15], and the ACL injury is, therefore, likely a multi-planar event. Prospective risk factor studies have shown conflicting results of whether the VM and/or the vertical ground reaction force (vGRF) can predict injury [9, 14]. Although female athletes are at greater risk of injury, our laboratory has reported that boys, and not girls, demonstrate greater VM [30]. Furthermore, the magnitude of forces has been shown to increase with greater training [31], whereas the incidence of ACL injury is greater in younger athletes who have had less training compared to the older athletes [36].

The ACL has been shown to mechanically weaken with fatigue [16]. Lipps et al. subjected cadaveric knees to repeated loading of 3 or 4 times the bodyweight during a simulated pivoting motion and found that a large proportion of the ACLs ruptured, with loading magnitude a key determinant to the number of load repetitions to failure [16]. Under these conditions, subject sex or tibial slope did not result in fewer cycles to failure; however, the researchers applied compressive and internal rotation loading concomitantly while also using relatively few loading cycles at greater loads compared to soccer match play [16]. If repeated multi-planar loading resulting in fatigue-failure of the ACL is the primary injury mechanism, the sex-dependent risk of ACL injury may be due to females having more frequent multi-planar loadings due to anatomic or neuromuscular factors rather than high magnitude of loading. The aim of this study was, therefore, to identify the timing of early peak VM, IRM, and vGRF for boys and girls during early stance, at the age where ACL injuries are beginning to occur [29]. The primary focus was to investigate whether differences would be found between male and female youth athletes with respect to the timing of peaks as well as the occurrence of coincident multi-planar loading. The primary hypothesis is that at this age, as it is not known if sex-dependent risk of injury has manifested, both sexes would have similar timing of peak forces. A secondary aim was to evaluate if the global stance-phase peak VM and vGRF, which have previously been linked to risk of ACL injury [9, 14], showed similar timing and co-occurrence as the early peak VM and vGRF. The secondary hypothesis is that these peaks would occur later in the movement and show less co-occurrence compared with the early peak forces.

## Materials and methods

This is a cross-sectional laboratory study investigating characteristics of a potential risk factor for ACL injury.

### Participants

Participants were recruited from five local soccer and team handball clubs. Recruitment and measurements took place between 2012 and 2014 spanning three consecutive seasons.

### Data collection

The study is from the baseline measurements of a prospective controlled trial. Each athlete performed five repetitions of the cutting maneuver on each leg, before and after a 5-min fatigue protocol, for a total of ten attempts per leg. Both fatigue conditions were pooled for this analysis. In addition to cutting maneuvers, biomechanical data collection included bilateral drop jumps and strength testing not included in this report. The methods of data collection are described in detail elsewhere [5]. An 8-camera motion capture system (Qualisys Corporation, Göteborg, Sweden) sampling at 200 Hz was used. Marker placement was performed by experienced physiotherapists who received hands-on training from the primary investigator (KB) to increase reliability. The reliability for 3D kinematic analysis has been established for a running task, similar to our protocol [32]. The accuracy of external marker-based kinematic analysis has been evaluated compared with radiostereometric analysis and found to be good for flexion–extension movements, but the error of measurements of rotations are large but systemic, increasingly so as flexion angles increase [35]. No study has compared marker-based kinematics and radiostereometric analysis during rotational movements such as the cutting maneuver, and the cutting maneuver mostly involves low flexion angles (< 50°). While the accuracy of the rotational estimates may be lacking, the reliability is still excellent and allows for between-group comparisons. The accuracy of positioning the ground reaction force vector has been reported to be around 0.5–1.0 cm which can result in around 7–14% errors when estimating the joint moments [21].

### Data processing

#### Selection of variables to study

In line with the multi-planar nature of ACL injuries, variables shown to produce strain on the ACL in cadaveric studies were assessed. All moments are reported as external moments, normalized by body mass. The variables are knee VM (N*m/kg), IRM (N*m/kg) [18], and vertical GRF (N/kg) [24].

#### Data synthesis

Recorded trials were digitized using the Qualisys track manager software (Qualisys AB, Göteborg, Sweden) and exported for further analysis in Visual-3D (C-motion Inc., Germantown, Maryland, USA). A pipeline of commands was programmed to identify the following events: (a) Initial contact (defined as the first frame with vGRF > 15N), (b) 100 ms after IC (defined as 20 frames after IC), (c) up to three peaks for each variable of interest between initial contact and 100 ms. The data were exported as ASCII files and converted into spreadsheet format using a custom made computer program. This method has an effective detection window of 85 ms, from 10 ms after IC until 95 ms after IC. Using excel, the highest early force peaks were sorted from the three peaks recorded. Using a three-peak system was necessary, as often each variable would display multiple local maxima, the largest of which could be any one of them and the use of the absolute highest value within the 100-ms window often resulted in high values occurring at exactly 100 ms and thus not accurately represented the time frame of ACL injury. The study was approved by the Icelandic National Bioethics Committee, approval code VSNb2012020011/03.07.

### Statistical analysis

Statistical analysis was done using the SAS statistical package (SAS Institute, Copenhagen, Denmark). A mixed-models ANOVA was used to compare sexes and control for fatigue conditions, limb-differences*, and repeated measurements. An alpha of 0.05 was used to determine statistical significance. A post-hoc power analysis was performed using G*Power [6], which showed that with an alpha = 0.05 and power of 0.8 the current study is powered to detect a Cohen’s *f* = 0.26. Results are reported as means with 95% confidence interval (CI) instead of standardized effect sizes because CIs are better to evaluate if the zero difference (force summation) is likely.

## Results

Data on 129 athletes (60% female) were processed and available for analysis, of whom two were excluded due to processing errors for a total of 127 athletes. Participants’ mean (SD) weight, height, and age was 41 (9) kg and 1.5 (0.01 m), 10.5 years (1.2), respectively. Out of 2540 trials, 153 were excluded due technical errors. A total of 2387 trials entered the analysis, including both limbs, and both pre- and post-fatigue trials. The following results are presented as means with 95% CI.

### Time to peak − time between peaks

The timing of the force peaks is reported in Table 1. Most importantly, females show later force peaks with less spread compared with males. The time between peaks is reported in Table 2; most importantly females show lower mean times between peaks compared with males for the VM and IRM peaks. There was no difference between sexes in time between global stance phase peak VM and vGRF [120 ms (125–110 ms) vs 110 ms (120–100 ms), n.s.].

**Table 1:** Time from initial contact with the ground to peak force

	Boys			Girls			Difference	
	Mean	95% CI		Mean	95% CI		Mean	*P* for difference
		Lower	Upper		Lower	Upper		
Early peak (ms)								
VM	32	30	33	37	36	38	− 5	< 0.001
IRM	36	35	37	38	37	39	− 2	0.029
vGRF	38	37	40	37	36	38	1	ns
Global peak (ms)								
VM	204	195	213	238	230	245	− 34	< 0.001
vGRF	96	88	98	119	115	124	− 23	< 0.001

Early peaks occur within 100 ms after initial contact with the ground. Global peaks are maximum of whole stance phase

*VM* Valgus moment, *vGRF* vertical Ground Reaction Force, *IRM* knee Internal Rotation Moment

**Table 2:** The difference in timing between force peaks during the first 100 ms after ground contact

Time between peaks (ms):	Boys			Girls			Difference	
	Mean	95% CI		Mean	95% CI		Mean	*P* for difference
		Lower	Upper		Lower	Upper		
VM and vGRF	− 7	− 9	− 5	− 1	− 2	1	− 6	< 0.001
VM and IRM	− 5	− 6	− 3	0	− 2	1	− 5	< 0.001
IRM and vGRF	2	1	4	0	− 1	2	2	n.s

*VM* Valgus moment, *vGRF* vertical Ground Reaction Force, *IRM* knee Internal Rotation Moment

## Discussion

The key findings of this study are that all three forces displayed peaks during the first 100 ms after IC with the ground, with differences between sexes where girls’ peaks occurred later than the boys and with a smaller time between peak forces compared with boys, but not between global stance-phase peak VM and vGRF. This is to our knowledge the first study to report a sex-dependent difference in the timing of these events. A multi-planar load of anterior tibial translation, VM, and IRM has been proposed as the mechanism of ACL rupture [28]. Prospective risk factor studies have hitherto reported single peak variables as risk factors, most notably VM and vGRF [9, 14], which we have shown here to occur later in the movement than the ACL injury [13]. The same studies have also used the drop landing test, while ACL injuries more commonly occur during pivoting movements [7] such as the movement used in the current trial. The results presented here show that early force peaks are more likely to result in multi-planar load events during the estimated time of ACL injuries than are the global stance phase peaks. Cadaveric studies using repeated loading have demonstrated a fatigue-effect on the ACL resulting in a clinically relevant rupture type [16]. Recent studies using fluoroscopy have found that neither VM [34] nor vGRF [25] correlate with anterior tibial translation, but that increased Quadriceps muscle demand leads to higher anterior tibial translation although within a safe range [25]. Combined with recent reports from our lab that show that boys, and not girls, demonstrate higher peak forces during the first 100 ms after IC, it stands to reason that these peaks are not useful risk factors.

The frequency with which VM, IRM, and vGRF coincide may be one factor that predisposes athletes to more frequent high loading on the ACL contributing to a fatigue-effect to a greater extent than these loads do in isolation. Our results show that girls have a lower time between peak forces, indicating a greater frequency of multi-planar loads occurring at the knee. The variable timing of forces can be the missing link between training load, force magnitudes, and ACL injury risk.

There are some limitations related to the data collection and analysis process. The global peak values are easy to identify but occur later in the movement than an ACL injury. To extract the relevant data points required a more complex data extraction system due to the inherent variability of the data. Often, there would be multiple early peaks detectable, out of which only the largest was used in the analysis. The method we used is successful in extracting the largest early peak, however, by doing so secondary peaks large enough to be relevant for ACL injury risk may have been missed.

The sampling frequency of 200 Hz used in this study results in a 5-ms interval between frames of measurements. The results we report here are at the extreme of what a 5-ms interval can detect, but as no prior study has looked at the timing of these events, the sampling rate was estimated to be sufficient. A higher sampling frequency is required to clarify the temporal relationships between these variables.

The task was performed without a running approach and, therefore, the force peaks involved are likely of a smaller magnitude than would be seen during a sporting event. This is evident in that the early vGRF in the current study averaged 17 N/kg whereas Leppanen et al. [14] reported an average of 18.5 N/kg (calculation based on the reported sample mean weight). When scaling the speed and power of the movement up, it is reasonable to make the assumption that all forces would scale in a similar fashion. We argue that this results in a systematic bias where at best all variables are scaled down, and at worst the early peak forces increase more in comparison to the global peaks. The temporal relationships between variables are likely not affected by the running start to the same extent as the forces, and the results as reported are unlikely to be affected by this bias.

## Conclusions

The primary results are that coincidental summation of early force peaks are more common in girls compared with boys aged 9–12 years. Timing of force peaks, most importantly the co-occurrence, may be a crucial link in a multi-planar loading event as the cause of ACL injury. Clinically, these results indicate that athletes at-risk for ACL injury, such as those previously injured, should limit their exposure to repetitive cutting movements, as although they may demonstrate unremarkable magnitudes of forces acting on the knee joint, the timing of those forces may be important in the risk of ACL injury.

## Abbreviations

VM: Knee valgus moment
IRM: Knee internal rotation moment
vGRF: Vertical ground reaction force
ACL: Anterior cruciate ligament
IC: Initial contact with the ground
